# *SsMet1* is a critical gene in methionine biosynthesis in *Sclerotinia sclerotiorum*

**DOI:** 10.3389/ffunb.2025.1563395

**Published:** 2025-05-22

**Authors:** Nickisha Pierre-Pierre, Wei Wei, Richard Manasseh, Michelle Mendoza, George J. Vandemark, Weidong Chen

**Affiliations:** ^1^ Plant Pathology, United States Department of Agriculture (USDA) Agricultural Research Service, Pullman, WA, United States; ^2^ Plant Pathology Department, Washington State University, Pullman, WA, United States; ^3^ College of Veterinary Medicine, Washington State University, Pullman, WA, United States

**Keywords:** methionine metabolism, fungal mutant, virulence, *Sclerotinia sclerotiorum*, sclerotia, reduced growth and development, stress response, mycelium

## Abstract

Methionine, a key sulfur-containing amino acid, is involved in various important functions in cellular metabolism. Genes that encode enzymes to catalyze steps of the methionine biosynthesis pathway are essential for survival of fungi. The *SsMet1* (SS1G_11000) gene in *Sclerotinia sclerotiorum* is an orthologue of *BcStr2*, a gene characterized in *Botrytis cinerea* that plays a key role in methionine biosynthesis. In this study, we characterized *SsMet1* in *S. sclerotiorum* by creating *SsMet1*-deletion mutants, *Met1–2* and *Met1-4*, using a split marker technique. The *SsMet1*-deletion mutants were unable to grow on minimal medium and did not produce sclerotia. Supplementation with methionine and homocysteine rescued the defects in mycelial growth, but not sclerotial development of the *SsMet1-*deletion mutants. These results indicate that *SsMet1*-deletion mutants are auxotrophic for methionine. In addition, the *SsMet1*-deletion mutants exhibited increased sensitivity to osmotic and oxidative stresses, cell wall-damaging agents, and thermal stress. The mutants were avirulent on detached bean leaves, but virulence was also restored with methionine supplementation in minimal media. All the defects were restored by genetic complementation of the mutant with wildtype *SsMet1* allele. The results of this study indicate that *SsMet1* plays a critical role in the regulation of various cellular processes in *S. sclerotiorum*.

## Introduction

*Sclerotinia sclerotiorum* is one of the most destructive plant pathogens. There are more than 60 names that have been used to describe the diseases it causes (Purdy, 1979), including white mold, watery soft rot and stem rot (Bolton et al., 2006). *S. sclerotiorum* is known to infect dicotyledonous crops such as sunflower, soybean, dry bean, dry pea and chickpea, but has also some monocotyledonous species such as onion and Chinese chive (Boland and Hall, 1994; Choi et al., 2017). *S. sclerotiorum*’s mechanism to attack host plants includes cell wall and middle lamella degrading enzymes, toxins, defense substances and rapidity of infection (Lumsden, 1979). Annual losses from *S. sclerotiorum* in the United States have exceeded $200 million due to a lack of high levels of host resistance (Bolton et al., 2006).

*S. sclerotiorum* is a robust pathogen that thrives under diverse environmental conditions and survives as sclerotia for four to five years and up to eight years in soil (Adams and Ayers, 1979; Bolton et al., 2006). Management of *S. sclerotiorum* is difficult due to the fungus targeting different stages of crop development; infection can cause damping-off of seedlings, stem rot, and soft rot of fruits and flowers (Bolton et al., 2006). Successful disease control usually requires implementation and integration of multiple management practices. In the past, cultural practices such as field burning to reduce the number of sclerotia in the soil (Hind-Lanoiselet et al., 2005; Brooks et al., 2018) were used. Currently, the disease is managed in fields of many crop species using applications of fungicides, which can have negative environmental implications (Leroux et al., 2002; Mir et al., 2023). However, in many cases, especially for high value crops or on highly specialized farms, these methods are insufficient for white mold control.

Because of the central role of methionine in metabolism (Jastrzębowska and Gabriel, 2015) and growth control in fungi, methionine biosynthesis inhibition by fungicides has been explored in several filamentous fungi (Masner et al., 1994; Fritz et al., 1997; Scott et al., 2020). Methionine (Met), a key sulfur-containing amino acid, is not only a key amino acid of proteins, but is also involved in many essential functions in cellular metabolism through its main derivative S-adenosylmethionine (SAM). The pathways of methionine biosynthesis have been characterized extensively in some plants, bacteria, and fungi (Jastrzębowska and Gabriel, 2015) but have not been reported in *Sclerotinia*.

Recently, targeted disruption of genes involved in the metabolism of cysteine and methionine in *Alternaria alternata*, *AaMetB*, *AaMetC* and *AaMetX*, have been investigated as these genes are required for vegetative growth, conidiation, and pathogenicity of *A. alternata* (Gai et al., 2021). Fungal mutants that lack *AaMetB*, *AaMetC*, or *AaMetX* exhibited developmental deficiencies such as decreased aerial hyphae and conidia production, and inability to grow on minimal media. However, the defects in cell growth and development of these mutants were restored by exogenous applications of methionine or homocysteine, but not cysteine. This restoration indicated that *AaMetB*, *AaMetC*, and *AaMetX* mutants are typical methionine auxotrophs rather than cysteine auxotrophs (Gai et al., 2021).

In the study on *B. cinerea*, Shao et al. (2016) observed that the deletion mutant *ΔBcStr2*, an orthologue of *Str2* encoding a cystathionine γ-synthase (CGS) in yeast *Saccharomyces cerevisiae*, exhibited a decrease in virulence on different plant tissues. They also found that *ΔBcStr2* produced significantly fewer conidia compared to the wildtype and complemented strains. The mutant strain also showed increased sensitivity to agents that damage the cell wall and increased sensitivity to osmotic stress, which led to reduced mycelial growth of the mutant strain in host tissue.

In *Magnaporthe oryzae*, the methionine synthase, MET6, catalyzes the last step of methionine biosynthesis. *MET6*-deletion mutants were obtained by targeted gene replacement (Saint-Macary et al., 2015). The *Δmet6* mutants were unable to grow on minimal media unless supplemented with methionine*. M. oryzae Δmet6* mutants were non-pathogenic on intact and wounded barley and rice leaves. These results indicated that the Met biosynthesis pathway may be essential for fungal development, survival, and pathogenicity (Saint-Macary et al., 2015).

In this study, we investigated the role of *SsMet1*, a gene hypothesized to be involved in methionine biosynthesis, in *S. sclerotiorum*. Thus, we generated gene deletion mutants of *SsMet1*, characterized the function of *SsMet1* in mutant strains to evaluate the role of *SsMet1* in the regulation of various cellular processes in *S. sclerotiorum*. The deletion of the methionine biosynthesis gene demonstrated its role in growth, virulence, sclerotial development, and response to environmental stresses.

## Experimental methods

### *SsMet1* sequence acquisition and phylogenetic analysis

First, the sequence of *SsMet1* was identified as an orthologue sequence to *BcStr2* of *B. cinerea* on EnsemblFungi (Yates et al., 2022; Martin et al., 2023). These two sequences as well as 15 additional sequences highlighted as orthologues of SS1G_11000 were obtained by EnsemblFungi (Yates et al., 2022; Martin et al., 2023) and on the Basic Local Alignment Search Tool (BLAST) (https://blast.ncbi.nlm.nih.gov/Blast.cgi; Sayers et al., 2022). We searched against the NCBI genome database with a cut-off E-value of 1e^–10^. The translated protein sequences were aligned using the National Center for Biotechnology Information multiple alignment tool (COBALT: Multiple Alignment Tool; Papadopoulos and Agarwala, 2007). For phylogenetic analysis, maximum-likelihood phylogenetic dendrograms were constructed using MEGA11 with 1000 bootstraps (Tamura et al., 2021).

### Knockout of *SsMet1* in *S. sclerotiorum*

**Figure 1:**
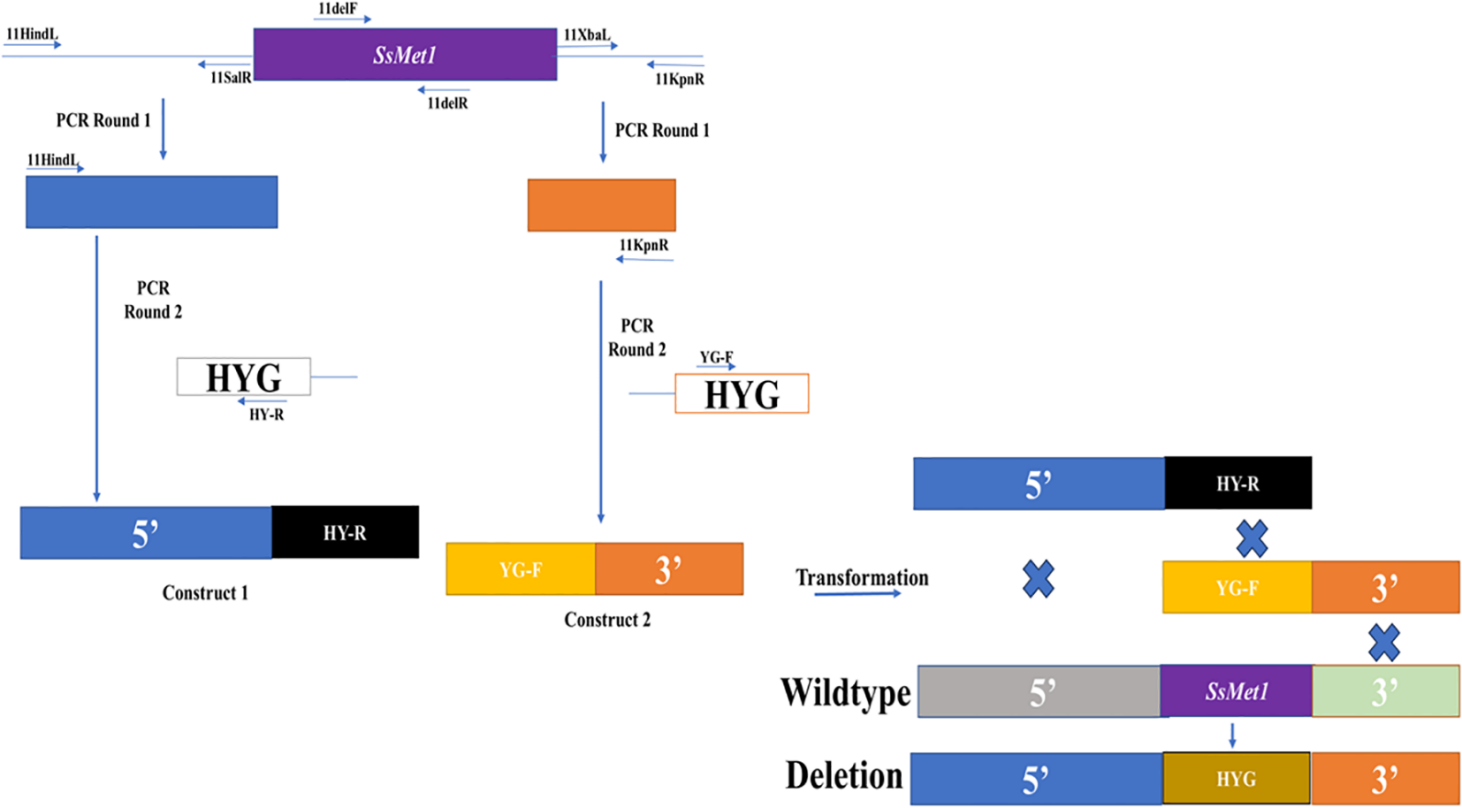
Knockout of the *SsMet1* gene in *S. sclerotiorum*. The schematic diagram illustrates the knockout strategy of *SsMet1* gene with the hygromycin-resistance gene (HYG) as the replacement (not drawn to scale).

Gene replacement mutants for *SsMet1* were created with a split-marker technique (Goswami, 2012). For the target gene, both the 5’ (840 bp) and the 3’ (507 bp) flanking sequences of the ORF were amplified from genomic DNA of the wildtype strain WMA1 (ATCC MYA-4521), with PCR primers 11HindL/11SalR and 11XbaL/11KpnR (**Table 1**) and cloned into the pJET1.2 cloning vector (Promega, Madison, Wisconsin) (**Figure 1**). The amplified 5’ and 3’ flanking sequences of *SsMet1* were then subcloned into pUCH18 (Lu et al., 1994) to generate pCU18-ORF-5’and pCU18-ORF-3’, which harbor a hygromycin-resistance gene (HYG) and the 5’ or 3’ flanking sequences at the 5’ and 3’ ends of the HYG gene (**Figure 1**). The 5’ and 3’ flanking sequences with their corresponding truncated HYG were amplified (**Figure 1**). Purified 5’-HY and YG-3’ fragments were then used to co-transform *S. sclerotiorum* protoplasts (Rollins, 2003). Transformants were selected and purified through repeated hyphal tipping on potato dextrose agar (PDA) containing hygromycin (100 ug/L). In the hygromycin-resistant transformants, the target gene was replaced by reconstituted HYG via homologous recombination mediated by the flanking sequences (**Figure 1**).

**Table 1:** PCR primers used in this study.

PCR primers	Sequence (5’ to 3’)	Purpose
11Hind-L	AAGCTTTCCTGGACGCCGATAGCGGATA	Knockout of *SsMet1*
11Sal-R	GTCGACCTGAGGGCTTTTTGGGTTT	Knockout of *SsMet1*
11Xba-L	TCTAGACGAATGGAGGTTATGGTGGA	Knockout of *SsMet1*
11Knp-R	GGTACCAAGGCTTCCAGGTTGTTAGTTC	Knockout of *SsMet1*
11-L	GAGCTCATGTCCGTCATCGAACTTGGAGAGT	Complementation of *SsMet1*
11-R	CCCGGGAGACGTTGAATCTGCCGCAGCTTTC	Complementation of *SsMet1*
11delF6	GAGAATGCAACGTCATGGGC	Confirmation of *SsMet1* allele
11delR6	ACCAAGGGATGGGATCGAAG	Confirmation of *SsMet1* allele

The deletion of *SsMet1* and gene replacement in *Met1–2* and *Met1–4* mutants were confirmed by rt-PCR assays with the 11delF6/11delR6 primers (**Table 1**). For rt-PCR, mycelia were harvested, freeze dried and total RNA was isolated using the RNeasy Plant Mini Kit (Qiagen, Germantown, MD), according to the manufacturer’s instructions. All RNA samples were diluted to 400 ng/µl and cDNA was synthesized using the 5X all-in-one RT Mastermix (Applied Biological Materials Inc., Richmond, Canada), according to the manufacturer’s instructions.

### Complementation of *SsMet1*

The *SsMet1*-deletion mutant was complemented with the full-length wildtype allele of *SsMet1* gene. The full-length *SsMet1* gene was amplified from the cDNA of the wildtype WMA1 strain with 11-L/11-R primers (**Table 1**). The PCR product was cloned into a pJET1.2 blunt cloning vector (Promega, Madison, Wisconsin) and digested with *SacI* and *SmaI*. The resulting *SacI-SmaI* fragment was cloned into a complementation vector (pCETNS) in the *Sac1/Sma1* sites and used for *Agrobacterium*-mediated transformation. Transformants were selected on PDA plates containing 150 μg/ml geneticin (G418; Ameresco, Ohio) for two rounds of hyphal tip transfers under geneticin selection, and the presence and expression of the wildtype *SsMet1* allele were confirmed by rt-PCR assay using primers 11delF6/11delR6 (**Table 1**).

### Characterization of *SsMet1-*deletion mutants

The *SsMet1*-deletion mutants were compared with the wildtype strain for growth rate and sensitivity to various stress agents. To compare their growth rates, colonized agar plugs (4 mm in diameter) of wildtype WMA1 and mutant strains *Met1–2* and *Met1–4* were taken from the edge of a 2-day-old culture, placed on the center of a fresh PDA plate, and incubated at 22°C. Cultures were monitored for three days for evaluation of colony morphology and diameter, and up to 30 days for sclerotial formation. Mycelial growth tests under various conditions were performed on minimal medium (MM) (10 mM K_2_HPO_4_, 10 mM KH_2_PO_4_, 4 mM (NH_4_)_2_SO_4_, 2.5 mM NaCl, 2 mM MgSO_4_, 0.45 mM CaCl_2_, 9 mM FeSO_4_. 7H_2_O, 10 mM glucose and 1 L water, pH 6.9) and MM amended with four different supplements: methionine (1 mM), cysteine (1 mM), homocysteine (0.5 mg/mL), or glutathione (0.5 mg/mL). Each plate was inoculated with a 4-mm-diameter mycelial-colonized agar plug taken from the edge of a 2-day-old colony on PDA. The diameter of the mycelium was measured with a digital caliper three days post inoculation. Each treatment was replicated three times for each strain and the experiment was repeated three times.

To test the sensitivity of each strain to various environmental stressors, colonized agar plugs (4 mm in diameter) taken from the margin of a 2-day-old colony on PDA were placed onto PDA plates amended with one the following six stressors: a cell wall stress agent, congo red (CR, 0.5 mg/mL); two osmotic stress generators, sodium chloride (NaCl, 1 M) and potassium chloride (KCl, 1 M); and three oxidative stress generators, sorbitol (1 M), hydrogen peroxide (H_2_O_2_, 10 mM) and calcofluor white (CFW, 0.5 g/liter). After incubation at 22°C for three days, the mycelial diameter in each plate was measured. Each experiment was repeated three times with three replications for each strain. The overall average of mycelia growth was obtained. Thermal stress sensitivity was tested by incubating 2-day-old colonies grown on PDA and then placed on fresh PDA plates under four temperature conditions (15, 20, 25, and 28°C) and measuring mycelial diameter after three days.

### Pathogenicity assays

Detached leaf disease assays on bean were conducted using mycelium of the wildtype and mutant strains grown on PDA. After a two-day growth on PDA, mycelium colonized agar plugs (4 mm in diameter) were placed on the leaves for observance of disease development. Lesion sizes were measured 48 hours post inoculation. This experiment was repeated three times with three replications for each strain. Supplementation of MM with methionine was also used to investigate pathogenicity restoration of the deletion mutants on detached been leaves. We inoculated the wildtype strain and deletion mutants on to MM amended with methionine and after two days of growth we used the fungal plugs to evaluate virulence on detached bean leaves.

### Statistical analysis

A linear model in conjunction with an analysis of variances (ANOVA) (R Core Team, 2020) were used to compare the mean growth rates of the wildtype, complement and mutant strains (*Met1–2* and *Met1-4*) inoculated on PDA and MM with amendments, which are the environmental stressors for PDA and the additions of methionine, homocysteine, cysteine and glutathione for MM. For the linear model and ANOVA, the fungus type and the media type were used as factors. The same approach was used to compare the growth responses to the different thermal stressors on PDA. For this analysis, the fungus type and temperature were used as factors. A linear model in conjunction with an ANOVA were also used to analyze the lesion sizes induced by the wildtype, complement and mutant strains on bean. For this analysis, the fungus type was used as a factor. The linear model provided the standard error. After each ANOVA, pairwise comparisons with Tukey’s HSD test were used to find means that were significantly different from each other. The alpha value of 0.05 was used for the ANOVA computation.

## Results

### Identification of *SsMet1* in *S. sclerotiorum*

*SsMet1* of *S. sclerotiorum* is an orthologue of *BcStr2* of *B. cinerea.* The homologous sequence for *BcStr2* gene from *B. cinerea* was found in *S. sclerotiorum* following a BLAST search against the NCBI genome database. The *SsMet1* protein shares 91.72% identity with that of *BcStr2*. Because of the high similarity of their amino acid sequences, *SsMet1* was identified as an orthologue of *BcStr2* in the *B. cinerea* genome database. We named this gene *SsMet1* due to *BcStr2* (an orthologue of *Saccharomyces cerevisiae*, *Str2*) being involved in methionine biosynthesis. The *SsMet1* gene is 1815 bp in length and encodes 604 amino acids. The structure of the nucleotide sequence was predicted to contain two introns of 96 and 71 bp located at positions 625 and 816 of the sequence, respectively. Analysis of the characteristic domains of the *SsMet1* protein with EnsemblFungi indicated that *SsMet1* contains a pyridoxal phosphate-dependent transferase domain (Yates et al., 2022; Martin et al., 2023).

### Sequence acquisition and phylogenetic analysis of *SsMet1* orthologues

**Figure 2:**
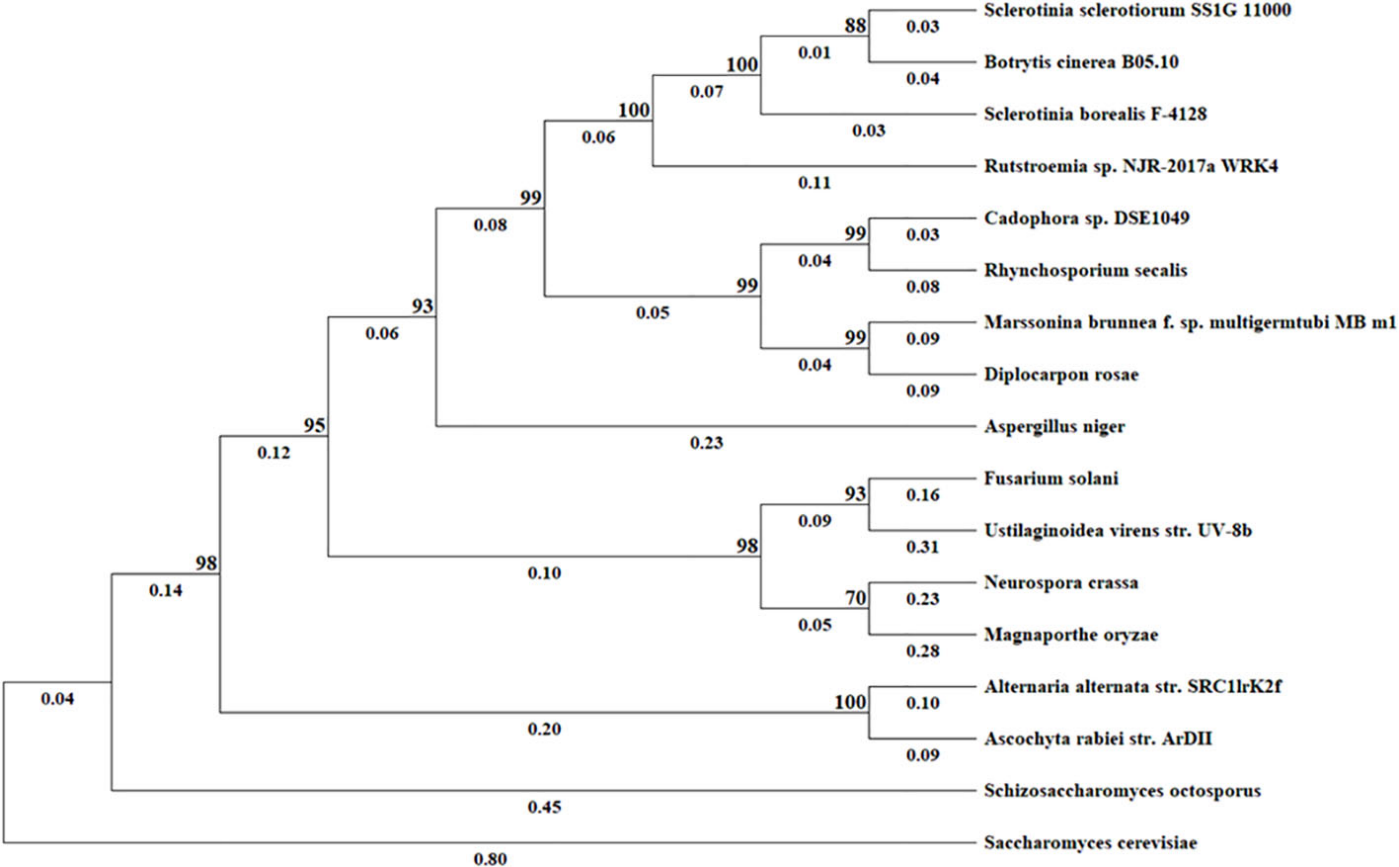
Evolutionary analysis by Maximum Likelihood method. The evolutionary history was inferred based on amino acid sequences using the Maximum Likelihood method and JTT matrix-based model. The tree with the highest log likelihood (–9941.71) is shown. The percentage of trees in which the associated taxa clustered together is shown next to the branches. Initial tree(s) for the heuristic search were obtained by applying the Neighbor-Joining method to a matrix of pairwise distances estimated using the JTT model. All positions containing gaps and missing data were excluded (complete deletion option). Evolutionary analyses were conducted in MEGA11.

BLAST search of the NCBI genome database and EnsemblFungi using the SS1G_11000 protein sequence as the query identified homologous sequences in 16 other fungi (including *BcStr2*). We found this protein sequence to be highly conserved among the 17 fungi compared, with amino acid identity varying from 37.25% to 91.89% (**Table 2**). *S. cerevisiae* was used as an outgroup and has the least identity at 37.25%. To further elaborate the phylogenetic relationships for this gene among the 17 fungal species, Maximum-likelihood phylogenetic dendrograms were constructed with MEGA11 (Tamura et al., 2021) (**Figure 2**). Regarding the protein sequence, the phylogenetic tree shows that *S. sclerotiorum* and *B. cinerea* are sister taxa. In addition, *S. sclerotiorum*, *B. cinerea*, *S. borealis* and *Rutstroemia* sp. NJR-2017a WRK4 form one monophyletic group. The taxa including *Cadophora* sp. DSE1049*, R. secalis*, *M. brunnea* and *D. rosae* form another monophyletic group and these two groups as a whole share a common ancestor. A third monophyletic group includes *F. solani*, *U. virens*, *N. crassa*, and *M. oryzae*. The tree also displays an ancestor further back in time that connects this monophyetic group to the previous two discussed that includes *S. sclerotiorum*. Lastly, the furthest ancestor highlighted in this tree shows that *S. sclerotiorum* and *S. cervisiae* share a distant common ancestor.

**Table 2:** Amino acid sequence identity.

Species	Amino Acid Identity %
*Sclerotinia sclerotiorum* 1980 UF-70	100
*Sclerotinia borealis* F-4128	91.89
*Botrytis cinerea* B05.10	91.72
*Rutstroemia* sp. NJR-2017a WRK4	80.53
*Cadophora* sp. DSE1049	75.00
*Rhynchosporium secalis*	72.94
*Marssonina brunnea* f. sp. multigermtubi MB ml	72.64
*Diplocarpon rosae*	72.44
*Aspergillus niger*	64.93
*Fusarium solani*	60.07
*Alternaria alternata* str. SRC1lrK2f	57.62
*Neurospora crassa*	57.17
*Magnaporthe oryzae*	57.10
*Ascochyta rabiei* str. ArDII	56.42
*Ustilaginoidea virens* str. UV-8b	54.62
*Schizosaccharomyces octosporus*	47.70
*Saccharomyces cerevisiae*	37.25

### Knockout and complementation of *SsMet1* in *S. sclerotiorum*

**Figure 3:**
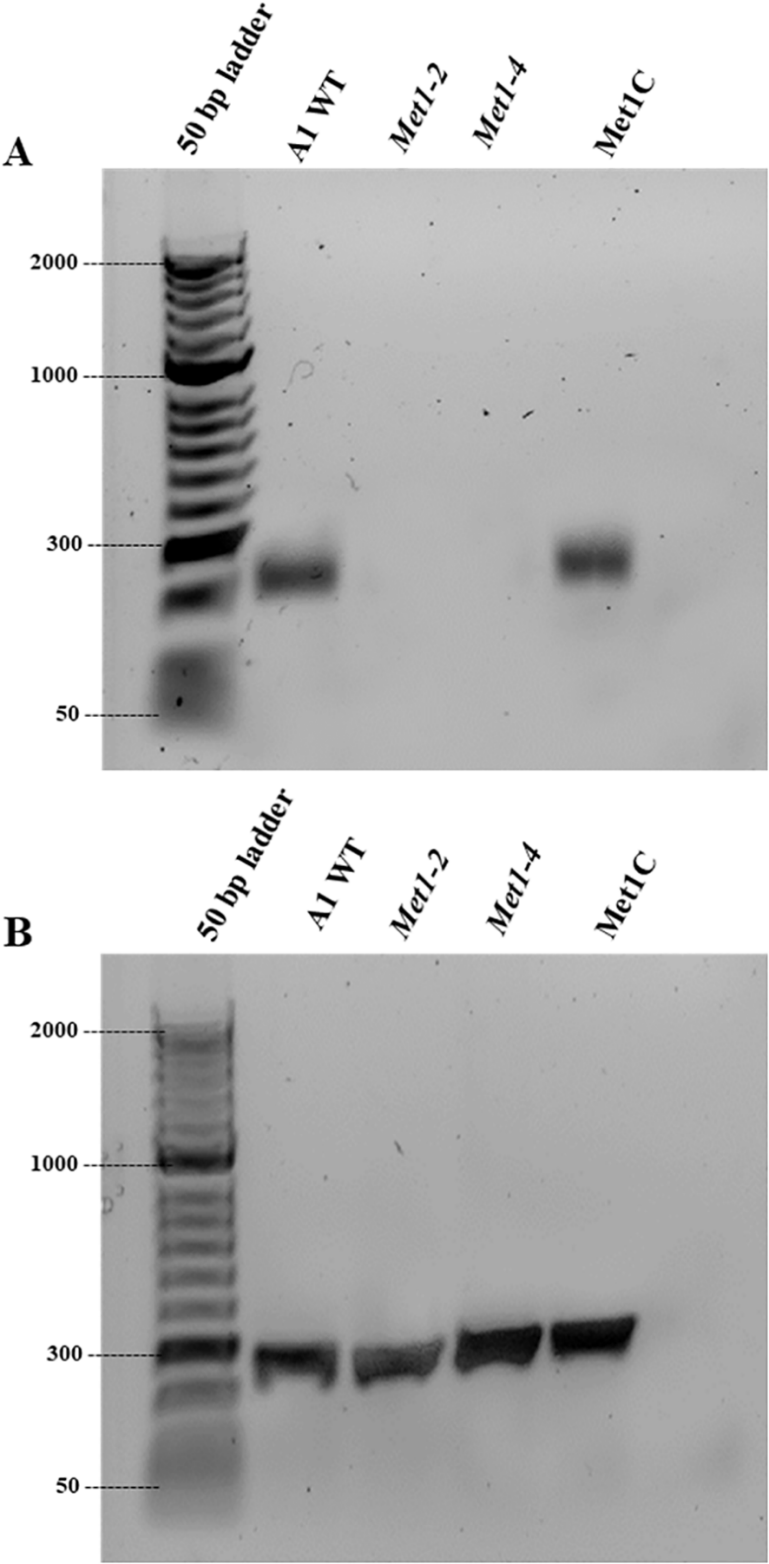
Verification of *SsMet1* knockout in *S. sclerotiorum* by PCR. **(A)** Confirmation of gene deletion of *Met1–2* and *Met1–4* using RT-PCR with *SsMet1* specific primers 11delF/11delR (217 bps). **(B)** PCR with SsGAR1 primers (NADPH-utilizing reductase) to check the RNA quality.

To confirm *SsMet1* gene knockout and complementation in *S. sclerotiorum*, rt-PCR was conducted with 11delF6/11delR6 primers (**Table 1**). The rt-PCR confirmed that the wildtype and complement strains of *S. sclerotiorum* have a band corresponding to the expected size of 217 bps, which was absent in the mutants (**Figure 3**), confirming that the *SsMet1* was deleted.

### Role of *SsMet1* in *S. sclerotiorum* pathogenicity

**Figure 4:**
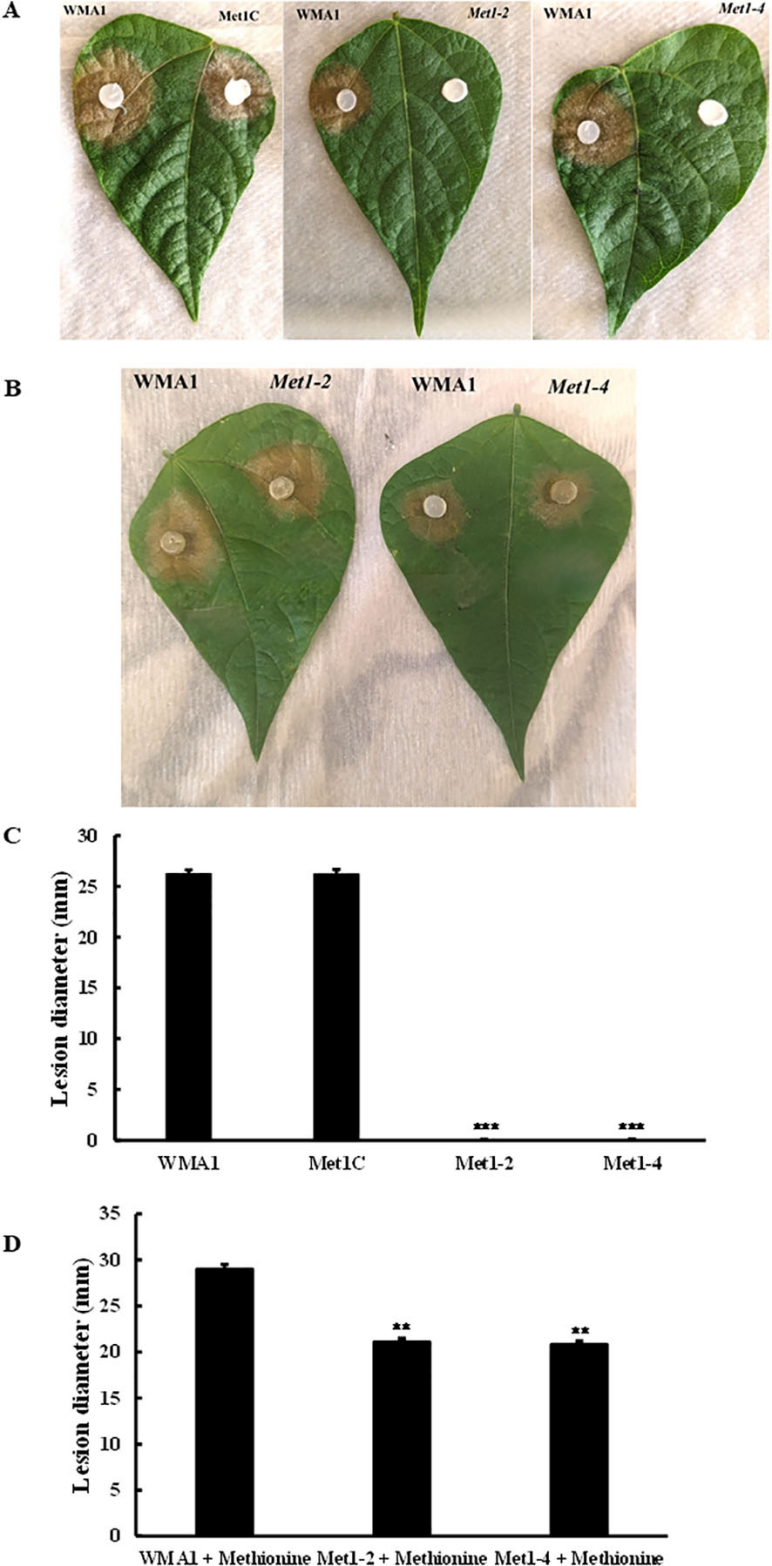
Pathogenicity assay of mutants in comparison to wildtype and complement strains. Pathogenicity assay on common bean leaves with **(A)** Wildtype, WMA1, complement, Met1C and *SsMet1*-deletion mutants, *Met1–2* and *Met1–4* grown on PDA. **(B)** WMA1 and mutants *Met1–2* and *Met1–4* after being inoculated on MM amended with methionine. Disease lesions were measured, and photos were taken 48h after inoculation. **(C, D)** Average lesion diameter. A one-way ANOVA in R and the Tukey HSD test were used to determine the significant differences of means from the wildtype, WMA1. The means of three replicates are presented and the error bars are standard errors. A significance threshold of 0.05 was used. Signif. codes: *** P < 0.001, ** P < 0.01.

To analyze the role of *SsMet1* in pathogenicity, *SsMet1* infection capability was evaluated on detached bean leaves. As shown in **Figure 4**, the wildtype and complement strains, WMA1 (average size of 26.3 mm) and Met1C (average size of 26.3 mm) respectively, caused disease lesions on detached leaves of bean 48 hours after inoculation. In contrast, the mycelia of *Met1–2* and *Met1–4* mutants were unable to infect bean leaves (**Figure 4A**). When the wildtype and deletion mutants were grown on MM amended with methionine and then used to inoculate detached bean leaves to observe virulence, the deletion mutants, *Met1–2* and *Met1–4* were able to cause disease on detached bean leaves (**Figure 4B**). The average disease lesion size for *Met1–2* and *Met1–4* were 21.1 mm and 20.8 mm respectively (**Figure 4D**).

### *SsMet1* is required for differentiation and sclerotial development

**Figure 5:**
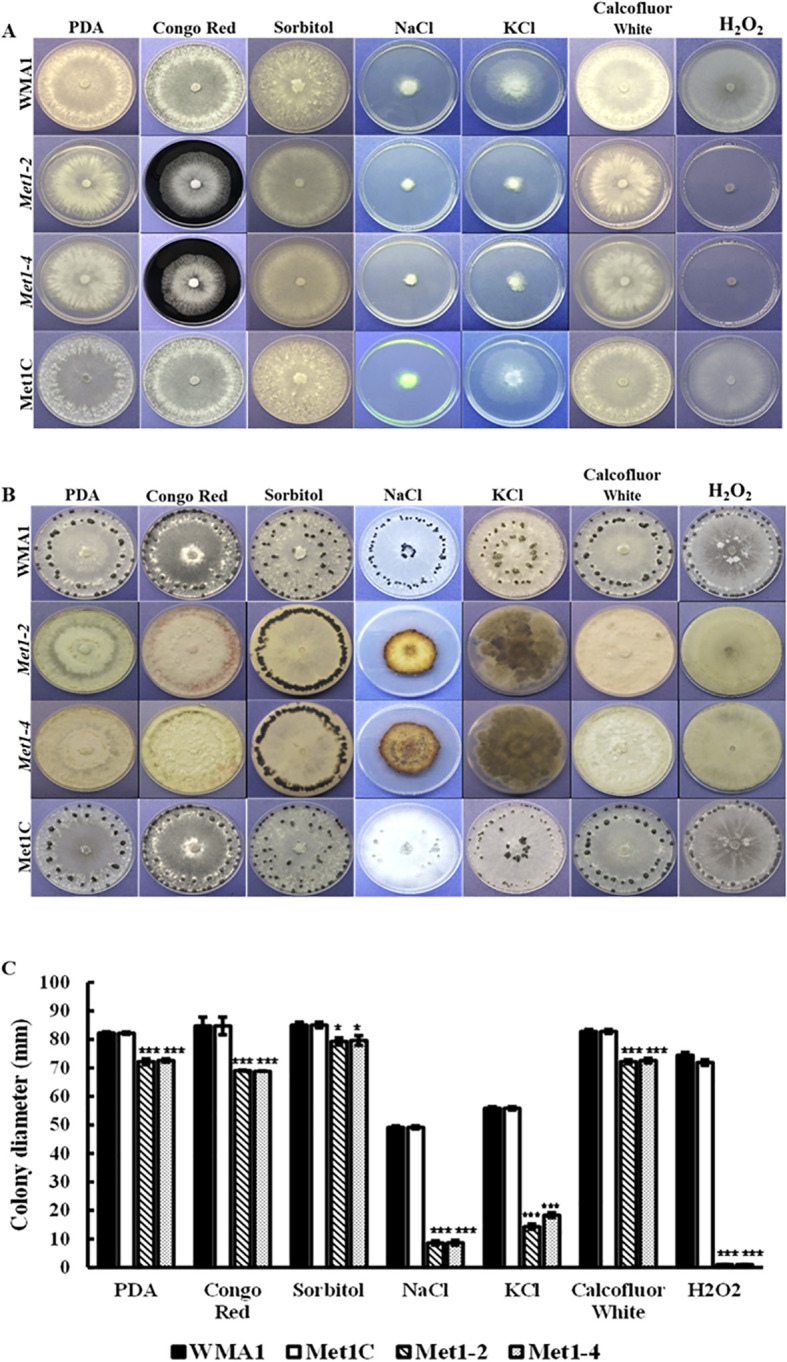
Sensitivity of the wildtype WMA1, complement Met1C and mutant strains *Met1–2* and *Met1–4* to cell wall-damaging, osmotic, and oxidative stress. **(A)** Comparisons of colony morphology on PDA plates amended with cell wall-damaging agent (0.5 mg/mL Congo Red), oxidative stress generators (1 M Sorbitol, 0.5 g/liter CFW and 10 mM H_2_O_2_) or osmotic stress agents (1 M NaCl and 1 M KCl). Photos were taken three days after incubation. **(B)** Sclerotial formation of the wildtype WMA1, *SsMet1*-deletion mutants *Met1–2* and *Met1-4*, and complement Met1C strains on PDA, and PDA amended with the above environmental stressors at 7 days and up to 30 days post inoculation. **(C)** Colony diameter. A one-way ANOVA in R and the Tukey HSD test were used to determine the significant differences of means from the wildtype, WMA1. The means of three replicates are presented and the error bars are standard errors. A significance threshold of 0.05 was used. Signif. codes: *** P < 0.001, * P < 0.05.

To study the growth of *SsMet1*-deletion mutants, *Met1–2* and *Met1-4*, wildtype WMA1 and the complement Met1C, were grown on PDA for three days at 22°C. The average colony diameter of WMA1 and Met1C strains were 82.2 mm and 82.4 mm, respectively. The *SsMet1*-deletion mutants *Met1–2* and *Met1–4* diameters were 72.2 mm and 72.6 mm respectively. The results show that the deletion mutants, *Met1–2* and *Met1-4*, grew slower on PDA than the wildtype and complemented strains, WMA1 and Met1C, respectively (**Figures 5A, C**).

**Figure 6:**
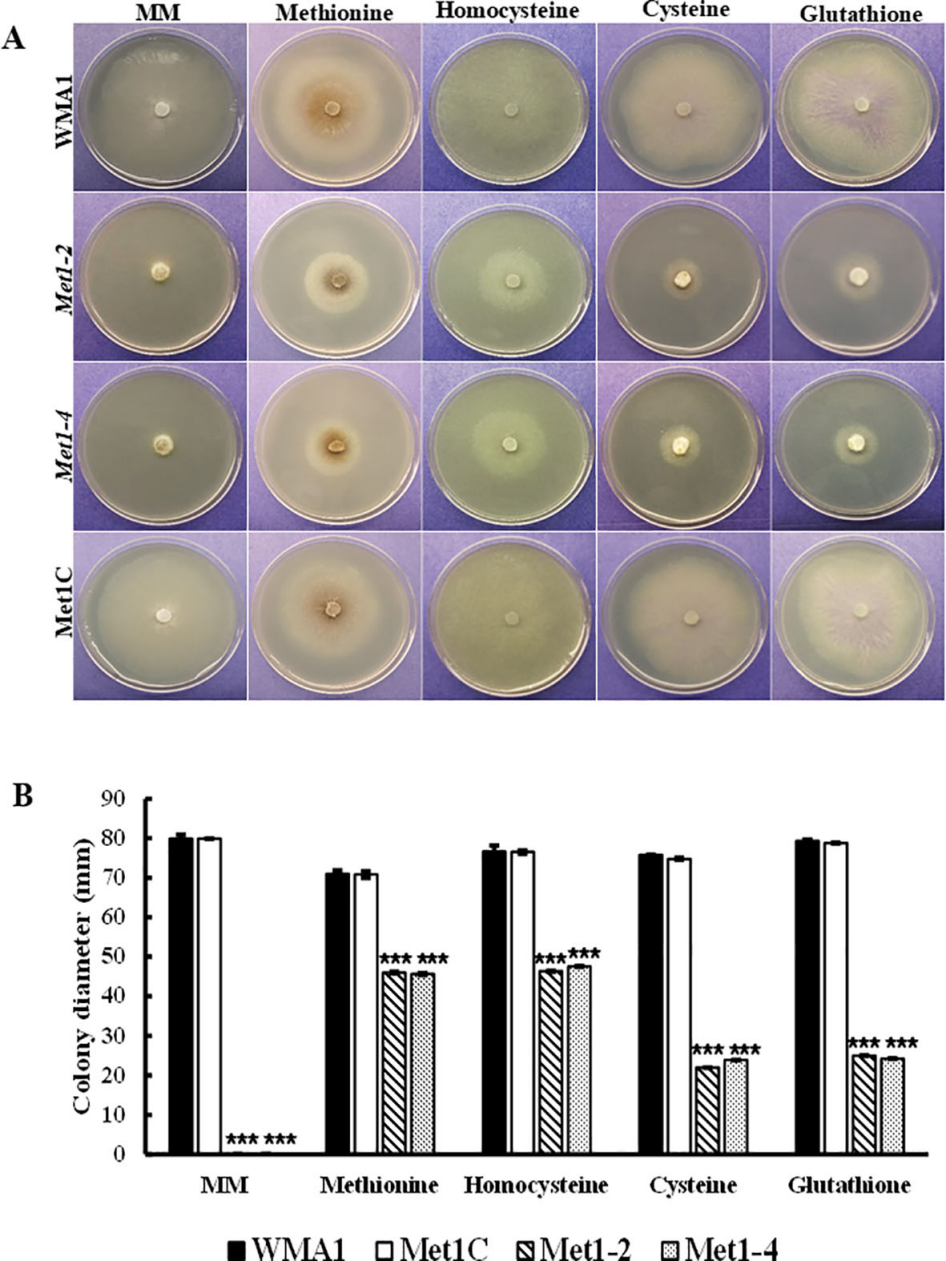
Chemical complementation assays of *SsMet1*-deletion mutants. **(A)** Mycelial growth of WMA1, mutant strains *Met1–2* and *Met1–4* and complement, Met1C on MM and MM amended with Methionine (1 mM), Homocysteine (0.5 mg/mL), Cysteine (1 mM) and Glutathione (0.5 mg/mL). **(B)** Colony diameter. A one-way ANOVA in R and the Tukey HSD test were used to determine the significant differences of means from wildtype, WMA1. The means of three replicates are presented and the error bars are standard errors. A significance threshold of 0.05 was used. Signif. codes: *** P < 0.001.

Naturally, we wanted to observe how sclerotial formation of the deletion mutants compared to the wildtype and complemented strains due to sclerotia being an important form of survival for *S. sclerotiorum*. Twelve days post inoculation on PDA, *Met1–2* and *Met1–4* mutants did not produce prominent sclerotia whereas the wildtype WMA1, and the complemented strain, Met1C, both produced normal sized sclerotia after seven days (**Figure 5B**). These results indicate that *SsMet1* is required for sclerotial development in *S. sclerotiorum*. In addition, the mutants were unable to grow on MM (**Figure 6A**), suggesting that *SsMet1* plays a role in mycelial growth and differentiation.

### *SsMet1* is crucial for stress response in *S. sclerotiorum*

**Figure 7:**
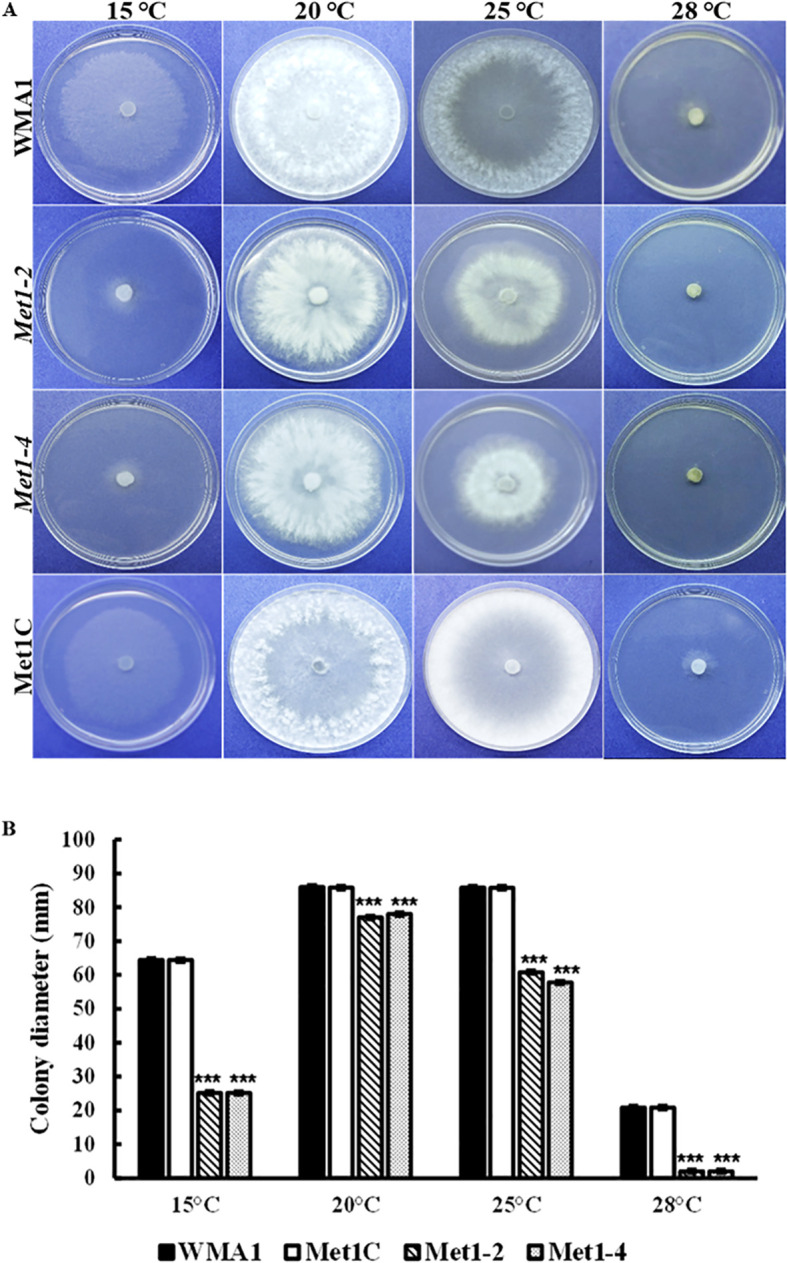
Sensitivity of WMA1, mutants *Met1–2* and *Met1–4* and complement strain Met1C to thermal stress. **(A)** Comparisons on PDA after incubation at 15, 20, 25 and 28°C for 3 days. **(B)** Colony diameter of wildtype, WMA1, complement, Met1C and *SsMet1-*deletion mutant strains *Met1–2* and *Met1-4*. A one-way ANOVA in R and the Tukey HSD test were used to determine the significant differences of means from the wildtype, WMA1. The means of three replicates are presented and the error bars are standard errors. A significance threshold of 0.05 was used. Signif. codes: *** P < 0.001.

To investigate the functions of *SsMet1* in *S. sclerotiorum* tolerance to environmental stresses, we observed the growth of wildtype WMA1, complement Met1C, and mutants *Met1–2* and *Met1–4* strains under conditions of osmotic and oxidative stresses, cell wall-damaging agents and thermal stress. To analyze the growth response associated with exposure to these environmental stressors the four strains were grown on PDA for two days at 22°C and then agar plugs from these 2-day-old cultures were inoculated onto PDA amended with the above listed stress agents stated in the methods section. Colony diameters were measured after three days of inoculation. Compared with the wildtype and complemented strains, *Met1–2* and *Met1–4* showed increased sensitivity to osmotic stress (reduced radial growth) generated by NaCl (1 M) (P < 0.001) and KCl (1 M) (P < 0.001), and the cell wall stress agent, congo red (0.5 mg/mL) (P < 0.001) (**Figures 5A, C**). *Met1–2* and *Met1–4* both exhibited increased sensitivity as shown by reduced growth to oxidative stress generated by H_2_O_2_ (10 mM) (P < 0.001), CFW (0.5 g/liter) (P < 0.001) and sorbitol (1 M) (P < 0.05) (**Figures 5A, C**). After fifteen days, the mutants were able to grow their mycelial to the edge of the PDA plate amended with H_2_O_2_. The initial growth size (measured after three days) for the *SsMet1*-deletion mutants was 1 mm and after 15 days the growth size increased to 82 mm. This increase in growth may be due to the instability of H_2_O_2_ and the loss of inhibitory effect over time. On exposure to thermal stress, the mutants showed sensitivity to temperatures both lower and higher than the optimal growth temperature of 20 °C (**Figure 7A**). At 15 °C and 28 °C, both *Met1–2* and *Met1–4* showed a significant decrease in growth compared to the wildtype after three days (**Figure 7B**).

### Mycelial growth restoration of *SsMet1* phenotype

Mycelium viability is very important for *S. sclerotiorum* growth. Minimal medium (MM) provides the minimal nutrients required for the fungus to grow. The mutants were not able to grow on MM because it lacks methionine. Our results show that in comparison with the wildtype (79.9 mm) and complemented (79.3 mm) strains, the *SsMet1*-deletion mutants, *Met1–2* and *Met1–4* were unable to grow on MM (0.0 mm) (**Figures 6A, B**). Supplementation with methionine (1 mM) and homocysteine (0.5 mg/mL) restored the growth defect of *SsMet1-*deletion mutants, *Met1–2* and *Met1-4*, on MM, although not to wildtype levels. The average mycelial growth for the wildtype, complement and mutants, *Met1–2* and *Met1–4* on MM amended with methionine were 71 mm, 70.8 mm, 46 mm, 45.6 mm respectively. The average mycelial growth for the wildtype, complement and mutants, *Met1–2* and *Met1–4* on MM amended with homocysteine were 76.6 mm, 76.5 mm, 46.4 mm, 47.5 mm respectively. Supplementation with cysteine (1 mM) and glutathione (0.5 mg/mL) also partially restored the growth defect of the *SsMet1*-deletion mutants, (**Figures 6A, B**). The average mycelial growth for the wildtype, complement and mutants, *Met1–2* and *Met1–4* on MM amended with cysteine were 75.8 mm, 74.8 mm, 22 mm, 23.9 mm respectively. The average mycelial growth for the wildtype, complement and mutants, *Met1–2* and *Met1–4* on MM amended with glutathione were 79.3 mm, 78.8 mm, 25 mm, 24.2 mm respectively. These results indicate that *SsMet1* is an auxotroph of methionine and methionine is required for processes like the initiation of translation, DNA synthesis, and the utilization of nutrients for optimal growth (Jastrzębowska and Gabriel, 2015).

## Discussion

### The biosynthesis of methionine

The methionine biosynthesis pathway has been studied in fungi (Saint-Macary et al., 2015; Shao et al., 2016; Gai et al., 2021). The main fungal route of methionine biosynthesis starts from homoserine, a branch point in the aspartate pathway leading to methionine or isoleucine through threonine production (Ejim et al., 2004). L-Homoserine first undergoes O-acetylation catalyzed by L-homoserine transacetylase Met2p. The sulfur atom is then introduced into the rising amino acid chain, either from inorganic sulfide via direct sulfhydrylation or from L-cysteine through the transsulfurylation pathway (Hébert et al., 2011; Kulikova et al., 2019). The sulfhydrylation reaction catalyzed by O-acetylhomoserine(thiol)-lyase Met15p leads to the conversion of O-acetylhomoserine to L-homoserine. Introduction of a sulfur atom through the transsulfurylation pathway requires the participation of two enzymes: cystathionine-γ-synthase (CGS) (Str2p) and cystathionine-β-lyase (Str3p). Str2p utilizes O-acetyl-homoserine and L-cysteine to produce L-cystathionine which, upon the action of cystathionine β-lyase Str3p, is converted to L-homocysteine. Finally, in all fungi, L-homocysteine is S-methylated by methionine synthase Met6p to generate methionine using 5-methyltetrahydrofolate as the methyl donor (Jastrzębowska and Gabriel, 2015; Shao et al., 2016).

### *SsMet1*-deletion mutants are methionine auxotrophic

In this study, we characterized *SsMet1*, a gene we believe that is implicated in methionine metabolism in *S. sclerotiorum.* We created *SsMet1*-deletion mutants, *Met1–2* and *Met1-4*. Methionine is a sulfur containing amino acid and plays a key role in cell proliferation, metabolism, protein synthesis and DNA methylation (Gai et al., 2019). The mutants grew slowly on PDA and were unable to grow on MM and did not produce sclerotia on MM. The sclerotia produced on PDA were abnormally small (**Figure 5B**). Supplementation with methionine and homocysteine to MM partially rescued the defects in mycelial growth, but not sclerotial development of the *SsMet1-*deletion mutants. These results indicate that the *SsMet1*-deletion mutants *Met1–2* and *Met1–4* are methionine auxotrophic, thus *SsMet1* is required for the regulation of cellular processes in *S. sclerotiorum* which includes amino acid biosynthesis and formation, and transferase activity. Thus, *SsMet1* is important for growth, pathogenicity, stress response and long-term survival. *SsMet1* is an orthologue of *BcStr2* found in *B. cinerea*. The *Str2* gene encodes a cystathionine γ‐synthase (CGS) that is a key enzyme in methionine biosynthesis in *Saccharomyces cerevisiae*. Therefore, CGS has been regarded as an attractive target for fungicide discovery (Leroux et al., 2002).

### *SsMet1* is necessary for virulence of *Sclerotinia sclerotiorum*

The *Met1–2* and *Met1–4* mutants were avirulent on detached bean leaves. This behavior is like that reported for the *ΔBcStr2* mutant in *B. cinerea*, which exhibited decreased virulence on host plants (Shao et al., 2016). Gai et al. (2021) showed that *AaMetB*, *AaMetC*, and *AaMetX* are required for full virulence in *A. alternata*. The defects in virulence of the *Met1–2* and *Met1–4* mutants on detached bean leaves were also restored by exogenous supply of methionine as reported by Gai et al. (2021). This suggests that *SsMet1* is essential for virulence of *S. sclerotiorum*.

### *SsMet1* is required for sclerotial formation of *S. sclerotiorum*

The *Met1–2* and *Met1–4* mutants were also unable to produce prominent sclerotial development. These results are consistent with *ΔBcStr2*, which exhibited decreased conidiation and impaired sclerotial development (Shao et al., 2016), whereas in *A. alternata*, mutants *AaMetB/MetC/MetX* showed defects in conidiation. We were able to restore the virulence of *Met1–2* and *Met1–4* by adding exogenous methionine to MM, but sclerotial development was not restored using the same method. This suggests that virulence and sclerotial development can be controlled by separate downstream factors.

### *SsMet1*-deletion mutants are more sensitive to stresses

The *Met1–2* and *Met1–4* mutants in the present study showed increased sensitivity to all environmental stresses. Both mutants grew slowly on sorbitol medium than the wildtype and complement strains but produced large sclerotia on PDA plates amended with 1 M sorbitol (**Figure 5B**), suggesting that exposure to sorbitol does not induce oxidative stress in these mutants to the point of not being able to produce resting structures. The mutants were not able to produce prominent sclerotia on PDA alone so the addition of sorbitol to PDA somehow reversed those effects. Growth was significantly reduced on PDA amended with H_2_O_2_ in the first 7 days, but both mutants were able to recover from the exposure to this H_2_O_2_ exposure and grow fully (15 days post inoculation), likely because H_2_O_2_ became ineffective with increased incubation time due to its short half-life. In addition, the deletion mutants, *Met1–2* and *Met1–4* were not able to produce sclerotia in the H_2_O_2_-amended medium. *AaMetR* was primarily sensitive to H_2_O_2_ and many reactive oxygen species (ROS)-generating compounds (Gai et al., 2021). The inactivation of *MetR* in *A. alternata* resulted in severe inhibition of methionine synthesis and increased sensitivity to H_2_O_2_ (Gai et al., 2021). In addition, *ΔBcStr2*, showed increased sensitivity to osmotic and oxidative stresses, cell-wall damaging agents and thermal stress (Shao et al., 2016). These results indicate that the *SsMet1*-deletion mutants were extremely affected by the environmental stressors and perhaps *SsMet1* is essential for ROS tolerance. The knockout and replacement of *SsMet1* has resulted in methionine metabolism being blocked. This can possibly affect the biosynthesis and expression of other metabolism related genes (Gai et al., 2021). In addition, *SsMet1* may interact with the genes that play a key role in cell wall synthesis and integrity, osmotic stress, and oxidative stress.

Methionine and homocysteine amendments were able to restore growth of the mutants on MM, but not to wildtype levels. Methionine supplementation to MM was able to restore virulence to the *Met1–2* and *Met1–4* mutants on detached bean leaves. In the research conducted by Das et al. (2024) exogenous methionine restored disease infection on rice and facilitated growth of *Rs_MET13*‐silenced *Rhizoctonia solani* gene in the presence of MM amended with 10 mM of H_2_O_2._ Homocysteine can be converted back to methionine through a process requiring folate, vitamin B12 and methionine synthase (McCaddon and Miller, 2023) and thus homocysteine was able to restore growth of the mutants to the same level as methionine on MM. We infer that homocysteine can also restore virulence to the *SsMet1*-deletion mutants. Glutathione and cysteine restored mycelial growth of the *SsMet1*-deletion mutants partially and in comparison to methionine and homocysteine, it was nearly 50% less after three days. Cysteine and glutathione cannot be converted back to methionine, resulting in poor growth restoration of the *SsMet1*-deletion mutants on MM. This slow rate of growth does indicate to us that virulence would be affected negatively and not be restored. This growth and virulence restoration indicates *Met1–2* and *Met1–4* are methionine auxotrophic mutants. In *A. alternata*, exogenous cysteine restored the growth and virulence defects of *AaMetR* suggesting that *AaMetR* is essential for cysteine biosynthesis (Gai et al., 2021). *SsMet1* is required for methionine synthesis and its deletion may prevent the initiation of other metabolic pathways such as cysteine biosynthesis due to it being described as a pyridoxal phosphate-dependent enzyme involved in Cys/Met metabolism (Martin et al., 2023). For *SsMet1* to be involved in Cys/Met metabolism (Martin et al., 2023), this allosteric dependence needs further investigation. In the transsulfuration pathway, homocysteine is converted to cysteine. Cysteine is then used to build glutathione. It is also important to note that fungi also have an alternative pathway (DUG pathway) to generate cysteine by degrading glutathione. In *S. sclerotiorum*, there are two genes, SS1G_04565 and SS1G_02111, related to the DUG pathway, DUG1 and DUG2 respectively. There is a possibility that the function of these genes can be dependent on *SsMet1* and in the absence of *SsMet1*, growth is not fully restored in the presence of exogenous cysteine and glutathione.

## Conclusion

Overall, we infer that methionine synthesis interacts with various biochemical pathways in *S. sclerotiorum*. The deletion of *SsMet1* demonstrated its role in growth, virulence, sclerotial development, and is important for the response to environmental stresses. *SsMet1* is a potential antifungal drug target in *S. sclerotiorum*. The *Str2* gene encodes a cystathionine γ‐synthase (CGS) that is a key enzyme in methionine biosynthesis in *Saccharomyces cerevisiae.* Therefore, CGS has been regarded as an attractive target for fungicide discovery. Further analysis can be done on the mutants for their sensitivity to fungicides. CGS is a proposed target of anilinopyrimidine (AP) fungicides (Leroux et al., 2002).

## Data Availability

The original contributions presented in the study are included in the article/supplementary material. Further inquiries can be directed to the corresponding authors.
